# Optimization of Au:CuO Thin Films by Plasma Surface Modification for High-Resolution LSPR Gas Sensing at Room Temperature

**DOI:** 10.3390/s22187043

**Published:** 2022-09-17

**Authors:** Manuela Proença, Marco S. Rodrigues, Diana I. Meira, M. Cidalia R. Castro, Pedro V. Rodrigues, Ana V. Machado, Eduardo Alves, Nuno P. Barradas, Joel Borges, Filipe Vaz

**Affiliations:** 1 Physics Center of Minho and Porto Universities (CF-UM-UP), University of Minho, Campus de Azurém, 4800-058 Guimarães, Portugal; 2 Instituto de Polímeros e Compósitos, Universidade do Minho, Campus de Azurém, 4800-058 Guimarães, Portugal; 3 IPFN, Instituto de Plasmas e Fusão Nuclear, Instituto Superior Técnico, Universidade de Lisboa, Estrada Nacional 10 Bobadela LRS, 2695-066 Lisboa, Portugal; 4 Centro de Ciências e Tecnologias Nucleares, Instituto Superior Técnico, Universidade de Lisboa, Estrada Nacional 10 Bobadela LRS, 2695-066 Lisboa, Portugal

**Keywords:** plasmonics, thin films, Au nanoparticles, nanostructural evolution, high-resolution LSPR spectroscopy, optical gas sensing

## Abstract

In this study, thin films composed of gold nanoparticles embedded in a copper oxide matrix (Au:CuO), manifesting Localized Surface Plasmon Resonance (LSPR) behavior, were produced by reactive DC magnetron sputtering and post-deposition in-air annealing. The effect of low-power Ar plasma etching on the surface properties of the plasmonic thin films was studied, envisaging its optimization as gas sensors. Thus, this work pretends to attain the maximum sensing response of the thin film system and to demonstrate its potential as a gas sensor. The results show that as Ar plasma treatment time increases, the host CuO matrix is etched while Au nanoparticles are uncovered, which leads to an enhancement of the sensitivity until a certain limit. Above such a time limit for plasma treatment, the CuO bonds are broken, and oxygen is removed from the film’s surface, resulting in a decrease in the gas sensing capabilities. Hence, the importance of the host matrix for the design of the LSPR sensor is also demonstrated. CuO not only provides stability and protection to the Au NPs but also promotes interactions between the thin film’s surface and the tested gases, thereby improving the nanocomposite film’s sensitivity. The optimized sensor sensitivity was estimated at 849 nm/RIU, which demonstrates that the Au-CuO thin films have the potential to be used as an LSPR platform for gas sensors.

## 1. Introduction

The colors in stained-glass windows and the dichroic effect of the famous Lycurgus Cup (4th century AD) are the result of the intense scattering and absorption of light from noble metal nanoparticles (NPs), which have attracted the interest of scientists for generations [1,2]. Recently, enormous research efforts have been focused on the understanding and using of the unique and tunable optical properties of NPs, which are associated with rapid progress in nanotechnology and nanoscale science, and have allowed the development of plasmonic-based nanocomposites for several exciting applications, including sensors [3,4,5]. The phenomenon behind these types of materials is related to the collective oscillations of conduction band electrons in metal NPs upon excitation with incident light at a wavelength of about one order of magnitude larger than the NP size [6,7]. Coupling of the incident light to the so-called localized surface plasmons in metal NPs leads to light extinction (absorption and scattering), and this phenomenon is called localized surface plasmon resonance (LSPR). Excitation of localized surface plasmons has two important consequences: electrical field enhancement near the NPs’ surface (rapidly decaying with the distance to the surface) and the occurrence of an extinction maximum at the plasmon resonant frequency [8]. For NPs such as gold (Au) and silver (Ag), this absorption band occurs at visible frequencies, and that is why they were so popular in ancient decorative applications [7,9,10,11].

The spectral shape and location of the LSPR band can be tailored by the shape, size, metal composition, and interparticle distance of NPs and by the refractive index (RI) of the environment surrounding the nanostructures [12,13,14,15]. These tunable optical properties allow nanostructures to be exploited for surface-enhanced spectroscopies [16], optical filters [17], plasmonic devices [18], and sensors [19]. Due to the nanometric size of NPs, the LSPR band position is very sensitive to the environmental dielectric properties, such as changes in liquid or gas density or even to any surface adsorption of chemical compounds [20,21,22]. When a target gas flows over the nanostructure, the RI changes, and consequently, an LSPR band shift occurs. Thus, the LSPR gas sensors’ response is based on measuring shifts of the LSPR band [4,23,24,25]. However, gas detection by LSPR sensing is a challenging task because inorganic gases such as Ar (η = 1.000281) and CO (η = 1.000338) exist as gases under ambient conditions, and their RIs are only different from the air by approximately 10^−4^ RI units (RIU), which may induce very small LSPR peak shifts, in the order of Δλ = 10^−2^ nm [24,26].

In order to make LSPR gas sensors functional, the sensor RI sensitivity (RIS) and the detection limits of sensors must be enhanced, by improving, for instance, the plasmonic nanocomposite design [27,28]. The structural uniformity of the nanocomposite and the NPs size distribution is important to achieve sharp LSPR peaks and, consequently, improve the LSPR-based sensor’s performance [29,30,31]. Furthermore, as NPs aspect ratio (width/height) increases and the symmetry of the NPs breaks, the electromagnetic field enhancement is improved, increasing the sensor sensitivity [32,33]. However, this involves precise manipulation and control of the NPs’ shape and size. The properties of the dielectric matrix, where the NPs may be hosted, also affect the sensitivity of plasmonic nanostructures [14,34,35].

In recent years, there have been several reports on the development of NPs-based plasmonic nanocomposites for gas sensing, in which NPs are coated with different dielectric materials, namely metal oxides [26,28,36,37,38]. In fact, metal oxides are widely used as chemoreceptive materials for gas sensing applications and, when combined with plasmonic NPs, result in an improved and reliable sensing platform [39,40]. However, due to the short decay length of localized surface plasmons, the sensing zone of the plasmonic nanocomposites is highly localized near the NPs [41,42], which limits the sensing volume to a few tens of nm from the surface of the NPs [43,44]. This means that the closer the NPs are to the target molecules, the greater their detection capabilities. Thus, to optimize the sensitivity of NPs embedded in metal oxides, physical [26,45] or chemical [46] etching processes must be used to semi-expose the NPs. However, the etching conditions must be carefully selected to avoid damaging the thin film sensor.

Recently, the authors developed a highly sensitive plasmonic nanomaterial composed of Au NPs embedded in copper oxide (CuO) matrix that can distinguish between different pure gases whose RIs vary by only 6 × 10^−5^ RIU [26,47]. However, the surface conditions that maximize the sensor sensitivity were not determined. Hence, the objective of this work is to enhance the sensing response of the Au:CuO thin film sensor by investigating the effect of different plasma etching treatments on the sensitivity and properties of the thin film. The effects of different Ar plasma treatments on the thin film chemical composition and morphology were studied and correlated with the gas detection results. The sensing capabilities of the films after different Ar plasma treatment times were investigated at room temperature by high-resolution LSPR spectroscopy, using He as the reference gas and Ar and 50 ppm CO:Ar as the test gases.

This work aimed to achieve the maximum sensing response of the Au:CuO thin film system, allowing it to be further exploited as a gas sensor hereafter or even to be used as an LSPR platform for label-free biosensors.

## 2. Materials and Methods

Nanocomposite thin films were produced by reactive DC magnetron sputtering using a rectangular Cu target (200 × 100 × 6 mm^3^, 99.99% purity) from Testbourne Ltd, Basingstoke, Hampshire, England, with gold pellets (several disks with a total surface area of 1200 mm^2^ and 0.5 mm thickness) symmetrically placed on the preferential sputtering zone. This target was mounted on the cathode of a custom-made vacuum chamber [48] at 7 cm from a grounded hexagonal substrate holder used in rotation mode (18 rpm), where SiO_2_ (fused silica) substrates from Neyco Vacuum & Materials, Vanves, France, were placed. The deposition was performed after a vacuum of approximately 3.8 × 10^−4^ Pa was reached inside the reactor. The vacuum system coupled to the deposition chamber is constituted by a primary rotary pump (AEG, model AMME 80ZCA4, Perugia, Italy) and by a turbomolecular pump (Adixen/Alcatel, model ATP 400, Annecy, France), responsible for the secondary vacuum. During the sputtering process, the atmosphere was composed of a mixture of Ar (flow of 15 sccm, partial pressure of 2.4 × 10^−1^ Pa) and O_2_ (flow of 14 sccm, partial pressure of 2.24 × 10^−1^ Pa) and the target was sputtered with a current density of 50 A.m^−2^ by a DC power supply (Hüttinger Elektronik, model PFG 2500DC, Freiburg, Germany) for 30 s.

**Figure 1:**
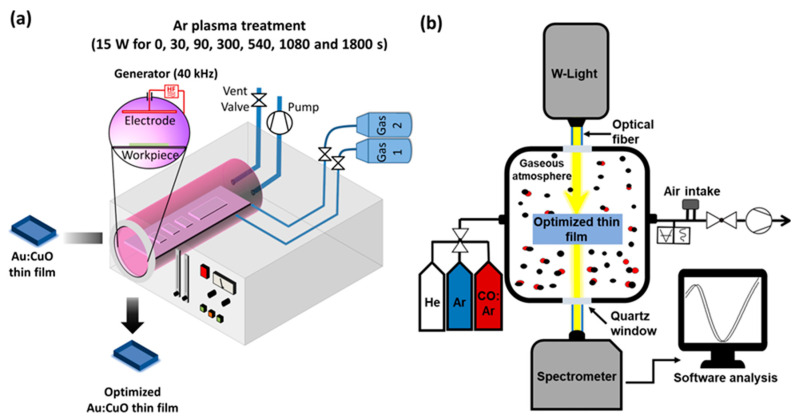
Simplified representation of the (**a**) Plasma Cleaner equipment used for Au:CuO film’s surface modification with different Ar plasma treatment times, and (**b**) custom-made high-resolution LSPR spectroscopy system for gas sensing test measurements in a controlled atmosphere.

Before the deposition, the surface of the SiO_2_ substrates was cleaned and activated by applying a plasma treatment of O_2_ (80 Pa, 5 min) followed by Ar (80 Pa, 15 min) using a Low-Pressure Plasma Cleaner by Diener Electronic (Zepto Model, Ebhausen, Germany) with a 13.56 MHz RF generator and a power of 50 W. The schematics of the Plasma system can be found in Figure 1a and the corresponding photograph in Figure S1a.

In order to induce the formation and growth of the Au NPs needed to tailor the thin film’s sensitivity, hence to find an LSPR band, a post-deposition in-air annealing procedure was carried out at 700 °C for 5 h in a Muffle Furnace (Nabertherm, model LE 6/11/R7, Lilienthal, Germany).

Different Ar plasma treatments were used to change the thin film’s properties and consequently maximize the nanoplasmonic film’s sensitivity. After the production of the plasmonic thin films, they were subjected to pre-selected Ar plasma treatment times (multiples of 30 s) in the Plasma Cleaner equipment above described, as illustrated in Figure 1a. The Ar plasma (80 Pa) was ignited with the power of the RF generator set to 15 W at room temperature.

In order to comprehend the influence of the etching time on the micro- and nanostructure of the films and its influence on the sensor sensitivity, the thin films were thoroughly characterized by different techniques. Thin film surface analyses were carried out by Rutherford backscattering spectrometry (RBS), X-ray Diffraction (XRD), Raman Spectroscopy, Atomic Force Microscopy (AFM), and Scanning Electron Microscopy (SEM).

RBS measurements were performed to evaluate the chemical composition of the Au:CuO films. A 2.5 MV Van Graaff accelerator (Model AN-2500 Type-A, High Voltage Engineering Europe, Amersfoort, the Netherlands) and a chamber with three detectors were used. One detector was placed at 140°, and two pin-diode detectors were located symmetrically to each other, both placed at a 165° scattering angle in an IBM geometry. Spectra were collected using 1.6 MeV ^4^He^+^. Selected samples were analyzed at an angle of incidence was 75° and detected at 160° in the Cornell geometry to enhance the depth resolution. The simulation model by Gurbich et al. [49] was implemented, and the RBS data were analyzed with the IBA DataFurnace NDF v10.0b [50].

In order to study the crystalline structure of the thin films, the samples were analyzed by XRD. A Bruker D8 Discover diffractometer (Karlsruhe, Germany) with Cu-Kα1 radiation (wavelength of 1.54060 nm) was used, operating in a grazing incidence mode at an angle of α = 2°. The diffractograms were recorded between 2θ angles from 30° to 100°, with a scanning step size of 0.025°.

The composition of phases present in the films was also evaluated by Raman spectroscopy. Room temperature Raman spectra were recorded on a LabRAM HR Evolution Raman spectrometer (Horiba Scientific, Villeneuve d’Ascq, France) coupled with Horiba Scientific’s Labspec 6 spectroscopy set, which provides not only a complete instrument control but also data processing. The Raman spectra of the samples were acquired with a 532 nm laser (Laser Quantum Torus 532, power 50–750 mW) in the range of 200–1200 cm^–1^ (acquisition time: 100 s; accumulations: 100; RTD time: 80; grating: 600 gr/mm; ND filter: 5%; hole: 150).

The effect of the plasma treatment on the thin film’s surface was investigated by Atomic Force Microscopy (AFM) before and after the plasma treatments. A high-resolution Nano-Observer AFM microscope from Concept Scientific Instruments (Les Ulis, France) was used in resonant mode with a 5 × 5 μm^2^ scan size, a resolution of 1024 × 1024 px, and 1 line/s scan speed. A silicon probe (ANSCM-PT-50) with Pt coating on both sides, a resonance frequency of 60 kHz, a spring constant of 3 N/m and a tip radius below 30 nm was coupled to the modular probe holder. The topography AFM data were analyzed using the freely available software Gwyddion (v. 2.59).

The morphology of the films was also studied by Scanning Electron Microscopy (SEM) in an Ultra-High Resolution Field Emission Gun Scanning Electron Microscopy (FEG-SEM), NOVA 200 Nano SEM, FEI Company, Hillsboro, Oregon, United States. Topographic images were performed at an acceleration voltage of 10 kV with a Secondary Electron (SE) detector. Atomic contrast images were realized with a Gaseous Analytical Detector (GAD) at an acceleration voltage of 15 kV. Then, a MATLAB algorithm was used to analyze the Au NPs size distributions, as well as the average Nearest Neighbor (N.N.) and Aspect Ratio (A.R.) of the NPs in the SEM micrographs with atomic contrast.

Gas sensitivity tests were conducted in a custom-made high-resolution LSPR spectroscopy system (schematics in Figure 1b and photograph in Figure S1b), described in more detail elsewhere [47], that allows real-time transmittance measurements in different atmospheres. The atmosphere surrounding the films was switched between pure He and pure Ar every 120 s for several cycles, at room temperature, while transmittance spectra were monitored in real-time. Then, similar tests were performed, but instead of pure Ar, a mixture of 50 ppm CO in Ar (50 ppm CO:Ar) was used. Before introducing the gases, the test chamber was kept under a primary vacuum at 1.8 × 10^2^ Pa, and each gas was introduced until a pressure of 2.6 × 10^4^ Pa was reached. For each gas atmosphere, a total number of 60 transmittance spectra were acquired by a modular spectrometer from Ocean Optics (HR4000 Model, Edinburgh, UK), giving a spectrum every 2 s. The transmittance spectra were then analyzed by the software NANOPTICS [51], which finds the wavelength peak position (minimum transmittance peak at the LSPR band) over time and calculates the average wavelength shift between the different gaseous atmospheres and the signal-to-noise ratio (SNR) of the measurements.

## 3. Results and Discussion

### 3.1. The Effect of Ar Plasma Etching on the Surface Properties of Au:CuO thin Films-Characterization of the Thin Films

Physical etching, using Ar plasma, is widely used to clean thin film surfaces, remove carbon contamination, and remove atoms or molecules from their composition [52,53,54]. Thus, Ar plasma treatment can affect the surface composition and surface morphology of the metal oxide thin films, leading to changes in their overall properties, including optical ones. Consequently, if plasmonic NPs are present, these changes in the metal oxide properties will strongly influence the dielectric environment, resulting in different LSPR spectra and sensing responses [55]. Therefore, it is necessary to study the chemical composition, structure, and morphology of the thin film according to the plasma treatment time to identify the optimal thin film design that is beneficial for sensing performance.

**Figure 2:**
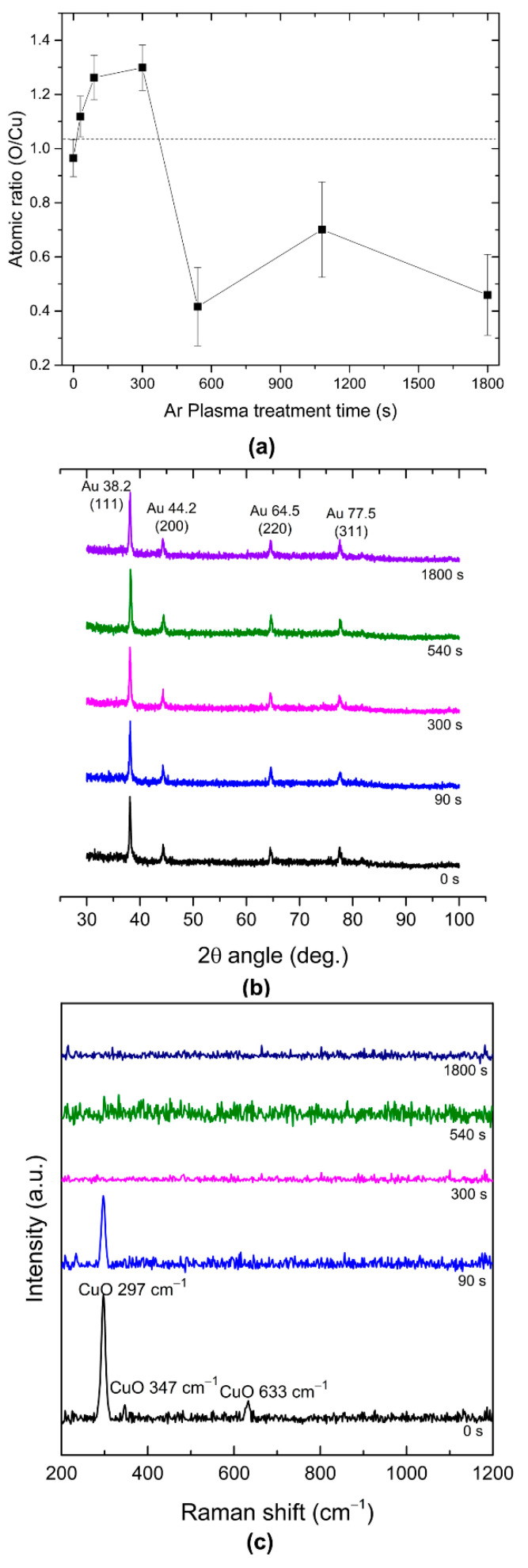
(**a**) O/Cu atomic ratio measured by RBS, (**b**) X-ray diffraction patterns, and (**c**) Raman spectra of the Au:CuO thin films as a function of Ar plasma treatment time.

The atomic composition of the Au:CuO thin film as a function of the plasma treatment time was investigated by RBS. According to the simulated RBS profiles, the Au concentration was estimated to be about 15.5 ± 0.5 at. %. Regarding the CuO matrix, its atomic ratio clearly changed as a function of the plasma etching time (Figure 2a). The simulation of the RBS profiles for Cu and O confirmed the formation of a CuO matrix very close to the stoichiometric condition, within the experimental error, up to 300 s of plasma treatment time. However, for longer plasma treatment times, there is a dramatic decrease in O content. This behavior is followed by a considerable increase in the surface roughness and measurement uncertainty, according to the best fitting curves obtained by RBS (Figure S2). This result is in agreement with other works [52,53,54], where it has already been demonstrated that Ar ion bombardment can easily break the metal-oxide bonds (Cu-O bonds in this case), thus removing oxygen from the film’s surface.

The crystalline structure of the Au:CuO thin film, after different Ar plasma treatment times, was also studied by XRD (Figure 2b). The diffraction patterns revealed Au crystallized in its most common structure, the face-centered cubic (FCC) [JCPDS file: 04–0784], characterized by diffraction peaks located at 2θ = 38.2°, 44.2°, 64.5°, and 77.5° of the (111), (200), (220), and (311) planes of the Au structure [15,56]. Furthermore, no diffraction peaks of other crystalline structures were detected, which means that CuO is probably in an amorphous phase, even after the thermal annealing treatment at 700 °C. Since the Au diffraction peaks of the samples remained rather similar as the plasma treatment time increased, this suggests that the plasma treatment did not cause significant changes in the Au NPs’ structure.

In order to confirm the amorphous nature of the oxide matrix, CuO thin films were prepared using the same experimental conditions as the Au:CuO films but without the inclusion of gold in the matrix. Different Ar plasma treatments were also applied to the CuO films to be analyzed by XRD (Figure S3). The diffraction patterns of the CuO thin films confirmed their amorphous phase. However, after 540 s of Ar plasma treatment, XRD patterns showed a very faint peak with low intensity at 2θ = 43.6°, which corresponds to the (111) plane of the FCC structure of metallic Cu [JCPDS file No. 04-0836] [57]. This may indicate that after 540 s of Ar plasma treatment, Cu crystalline grains may appear, due to oxygen amount reduction in the film, as verified by the composition analysis by RBS. Nevertheless, the identified XRD peak in the CuO film was not perceivable in the Au:CuO X-ray diffraction patterns.

Apart from the XRD analysis discussed above, Raman spectroscopy provides useful information about the chemical structure and molecular interactions of materials and might help to detect the existence of different oxide phases in the thin film. Raman scattering tests on the Au:CuO thin films with different Ar plasma treatments were performed at room temperature within the spectral region of 200–1200 cm^−1^. The respective Raman spectra are shown in Figure 2c. The three peaks observed before the plasma treatments (t = 0 s) are assigned to three Raman active modes (A_g_ + 2B_g_) of CuO. They are located at A_g_ (297 cm^−1^), B_g(1)_ (347 cm^−1^), and B_g(2)_ (633 cm^−1^) [58,59]. This result indicates the presence of a CuO phase, despite the fact that the XRD result had not shown any trace of crystalline domains (Figure 2b). This is because Raman spectroscopy is much more surface sensitive than XRD, and the latter can only detect crystalline grains of sufficient size of the oxide material, whereas the Raman technique is also capable of detecting the amorphous states, thus giving more surface-related information [60]. Therefore, based on the Raman and the XRD analyses, it can be concluded that both Au and CuO phases coexist within the thin film before the plasma treatments. Nonetheless, the intensity of indicated A_g_ and 2B_g_ peaks decreased with the plasma treatment time until 90 s of plasma treatment. After 300 s of plasma treatment, no peak was detected. The same trend was observed in the Raman spectra of CuO thin films (Figure S4). This behavior is related to the progressive vanishing of the CuO phase as plasma treatment time increases, due to the release of oxygen atoms from the matrix and the probable progressive formation of Cu grains, as aforementioned.

The performance of a gas sensor is greatly affected by the chemical composition and microstructure of the thin film’s surface since it depends on the interaction between its surface and the targeted molecules [28]. Thus, the understanding of favorable surface morphologies in the interaction with gases is important for the further improvement of gas sensors.

**Figure 3:**
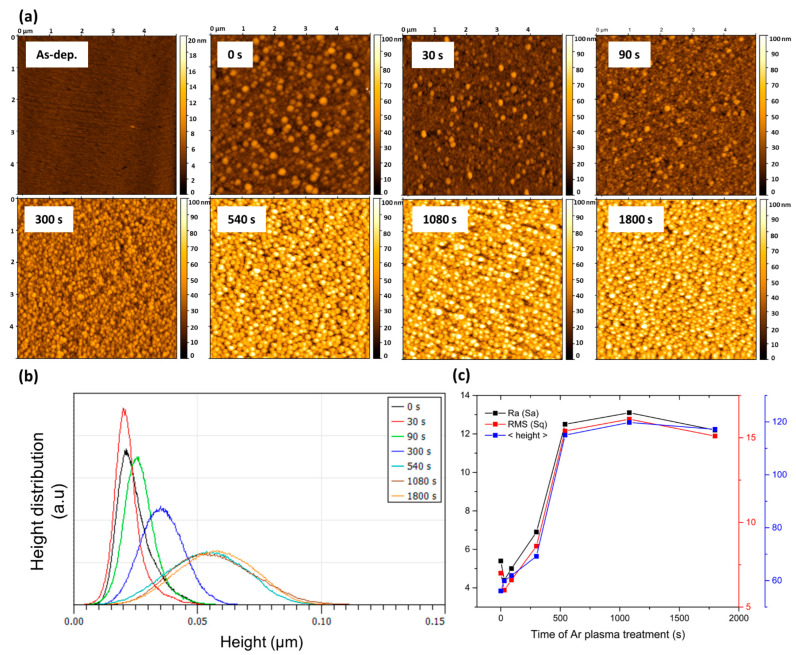
(**a**)Two-dimensional AFM height images, (**b**) height distribution profiles (AFM analysis using the Gwyddion software), and (**c**) surface mean roughness (Sa), root mean square roughness (Sq), and mean height (<height>) of the Au:CuO thin films as a function of Ar plasma treatment time.

The effect of Ar plasma treatment on the Au:CuO thin film surface was studied through AFM, and the corresponding 2D height images, analyzed in the scanning area of 5 × 5 μm^2^, are displayed in Figure 3a. First, the existence of many grain-like structures at the surface of the analyzed samples (t = 0–1800 s) is evident due to the clear presence of hills (peaks) and valleys in contrast with the smooth as-deposited sample (sample without annealing) [61]. Similarly, the same result was also verified in 2D AFM height images of CuO thin films (Figure S4), yet at lower peak heights, thus confirming a granular microstructure of the CuO matrix.

As plasma treatment time increases, more and more hills and fewer valleys are observed in Figure 3a. Roughness and height parameters were estimated from the analysis of the topography scans of the thin films’ surface after the different Ar plasma treatment times (Table 1 and Figure 3b,c). From the estimated parameters, two distinct behaviors are possible to observe. Up to 300 s of plasma treatment, the surface mean roughness (Sa) of the Au:CuO films, as well as the root mean square roughness (Sq), suffered changes slightly, varying from 5.4 to 6.9 nm and from 7.0 to 8.6 nm, respectively (Figure 3c and Table 1). Concerning the height distribution, which was in the range of 0–50 nm before de plasma treatment (Figure 3b), it slightly increased for smaller grains at 30 s of plasma treatment. This result is probably due to the removal of the top layers composed of CuO and hydrocarbons, present at the thin film surface, as verified in previous work [62], and the uncovering of some embedded NPs at the film’s surface. After 90 s and 300 s of plasma treatment, the height distribution (Figure 3b) became broader for higher grains and the mean height (<height>) increased (Figure 3c and Table 1) from 56.1 nm (t = 0 s) to 69.2 nm (t = 300 s). This behavior is certainly related to the CuO etching, as verified above by Raman spectra (Figure 2c), and to the partial uncovering of the Au NPs to free space.

On the other hand, as the plasma treatment time further increases, the surface mean roughness and the root mean square roughness (Figure 3 and Table 1) significantly increase to 12.2 (t = 540 s) and 15.1 nm (t = 1800 s), respectively. Furthermore, the height distribution became broader (Figure 3b), with sizes in the range from 30 to 100 nm, mainly due to the appearance of a granular microstructure throughout the entire surface. The mean height (Figure 3c and Table 1) also notably increased to 115 nm (t = 540 s) and 117.2 nm (t = 1800 s). This emergence of higher peaks is certainly related to the oxygen reduction in the films and the consequent formation of under-stoichiometric CuO nanostructures after the 540 s of plasma treatment (Figure 2a). The same effect was also verified in the 2D AFM height images of CuO thin films (Figure S5a), where a noticeable increase in the roughness and higher peaks (Figure S5b,c) are visible after the 540 s of Ar plasma treatment. Indeed, the AFM analysis of Au:CuO (Figure 3) and CuO (Figure S5) thin films are similar after the 540 s of Ar plasma treatment, which confirms that the Ar plasma treatment particularly affects the CuO matrix rather than Au NPs, leading to the appearance of nanostructures, perhaps CuOx or even O-doped Cu phases, with dimensions comparable to the Au NPs.

**Figure 4:**
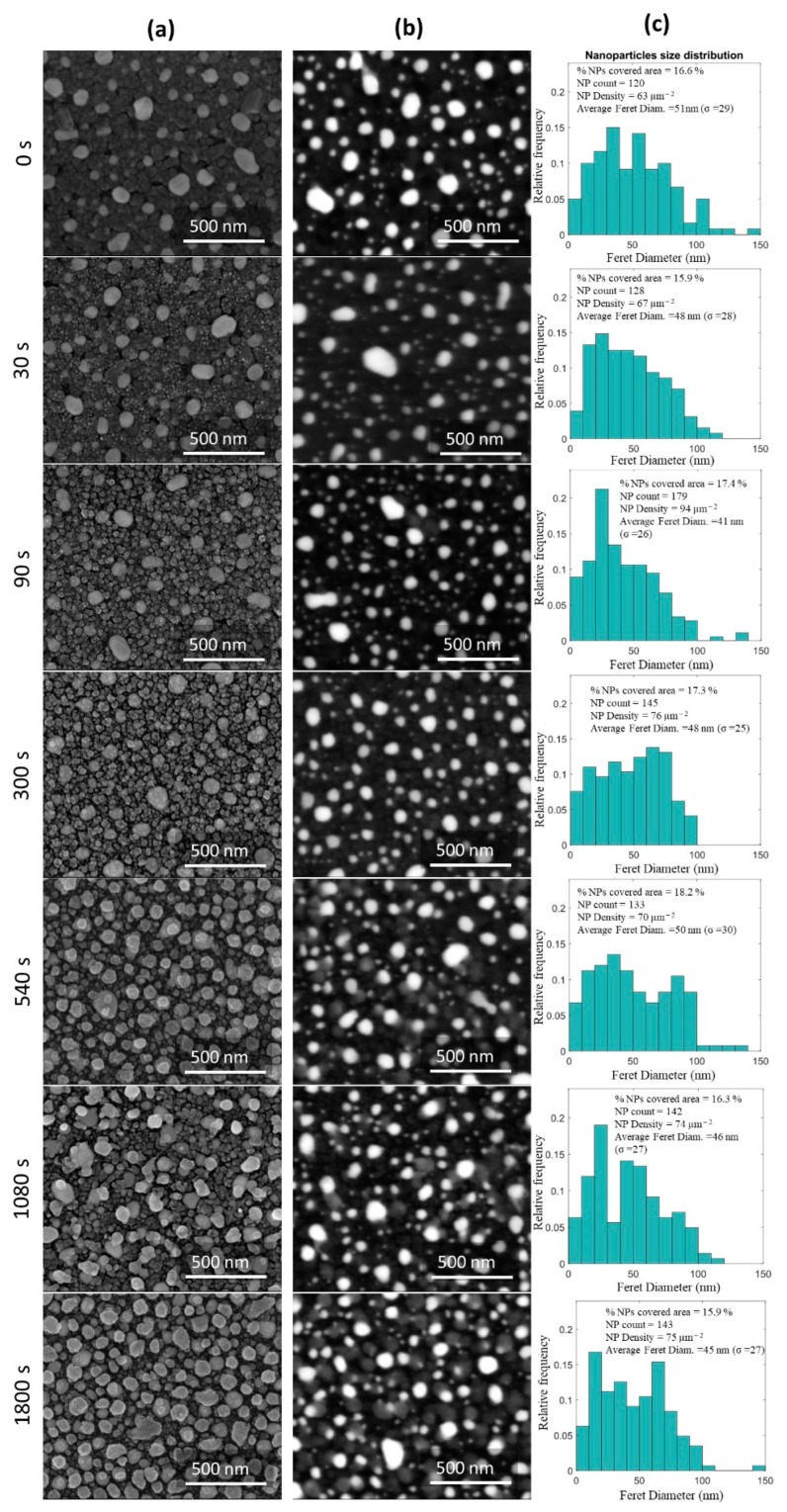
SEM micrographs of the Au:CuO thin films as a function of Ar plasma treatment time observed in top-view using (**a**) secondary (SE) and (**b**) gaseous analytical electron detectors with atomic weight contrast. The size distribution histograms of the Au NPs present in the micrographs with atomic weight contrast are displayed in (**c**).

The surface morphology of the films was also studied by SEM and observed in top-view using a secondary electron detector (SE) and atomic weight contrast with a Gaseous Analytical Detector (GAD). The corresponding SEM micrographs are represented in Figure 4a,b, respectively, as a function of the plasma treatment time. The Au NPs present in the micrographs with atomic weight contrast were analyzed by a MATLAB algorithm, and the results are shown in Figure 4c and Table 1.

The effect of the Ar plasma treatment time in the CuO matrix is perfectly visible in Figure 4a, while its consequence in Au NPs (white spots) is possible to be verified in Figure 4b. As expected, a significant number of roughly spherical Au NPs dispersed in the CuO matrix is noticeable at the thin film’s surface before the plasma treatment (t = 0 s) [15]. After 30 s of plasma treatment, the morphology of the host CuO matrix was changed. It became smoother, and some bright dots appeared on its surface (Figure 4a). This result seems to correlate with the AFM analysis, confirming that some Au NPs embedded in the CuO matrix were uncovered or, at least, were closer to the surface due to top layer removal. However, after 90 s of plasma treatment, the CuO matrix became more granular (Figure 4a), and the number of NPs at the surface slightly increased from 120 to 179 (Table 1). The NPs size distribution became somewhat narrower (Figure 4c), probably due to the appearance of extra and smaller Au NPs perceivable at the surface. This led to the decrease in their average size (<Feret diameter>) from 51 to 41 nm and the shortening of the average distance between the NPs (<Nearest Neighbor>), as can be seen in Table 1. Thus, CuO etching after 90 s of plasma treatment can be confirmed, as above verified by Raman and AFM analyses (Figure 2c and Figure 3, respectively). After 300 s of plasma treatment, the CuO etching becomes more evident (Figure 4a). However, no considerable changes are visible in the Au NPs distribution at 300 s and for longer plasma treatment times.

After 540 s of plasma treatment, the host CuO matrix became increasingly granular (Figure 4a), with sizes comparable to the Au NPs. Here, the presence of gray spots that can be related to O-doped Cu nanostructures is observed, confirming all the above-mentioned assumptions. Since Au has an atomic weight higher than Cu, the first one backscatters electrons more efficiently than the latter, distinguishing them by different brightness intensities [63]. Once again, these results confirm that the Ar plasma treatment particularly affects the CuO matrix.

**Figure 5:**
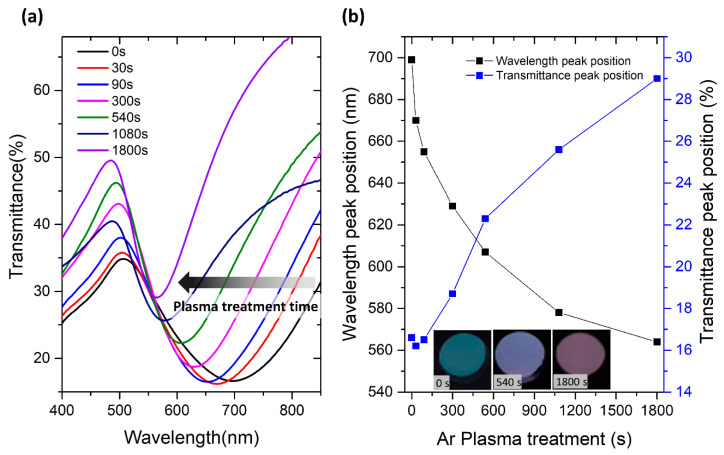
Transmittance spectra (**a**) of the Au:CuO thin films after different etching times with Ar plasma and wavelength and transmittance peak position (**b**) of the Au:CuO thin films as a function of Ar plasma treatment time, with inset of pictures of the Au:CuO thin films in transmittance after 0, 540, and 1800 s of Ar plasma.

The chemical composition and morphology developed by the Au:CuO thin films for different Ar plasma treatments are essential factors that influence the optical properties of the films and, consequently, their LSPR sensing performance. The LSPR bands of the films for different plasma treatments were scanned via transmittance optical analysis, whose spectra are shown in Figure 5a.

As expected, the transmittance spectrum of the plasmonic thin film varies according to the plasma treatment time applied. As the etching time increased, the LSPR band became narrower, and the wavelength peak position blue shifted. It decreased from the initial 699 nm (t = 0 s) to 629, 607, and 564 nm after 300, 540, and 1800 s selective plasma treatment times, respectively (Figure 5b). On the other hand, the transmittance peak position of the LSPR band significantly increased with increasing etching time for periods longer than 90 s. It increased from 16.6% (t = 0 s) to 29% after 1800 s of etching (Figure 5b). These changes in the LSPR bands upon Ar plasma treatments are also perceptible as a color change (Figure 5b-as insets). The color of the film is blue up to 300 s of plasma treatment, turning violet after plasma treatment of 540 s, and then pink at t = 1800 s. Certainly, those variations are related to the decrease in the RI of the medium surrounding the NPs with increasing surface treatment time [9,27,64]. In fact, before the Ar plasma treatment (t = 0 s), the Au NPs of the thin film are already partially embedded in the CuO matrix, while the remaining part is air-exposed. Until the 90 s of Ar plasma treatment, the wavelength peak position blue-shifted but did not significantly change its transmittance peak position, probably due to the removal of only the top layers of the film. After that time of plasma treatment, CuO erosion is suggested. The CuO matrix becomes thinner and less dense, which leads to a significant transmittance increase in the LSPR bands after 300 s of plasma treatment. Furthermore, the formation of O-doped Cu nanostructures is once again confirmed by the transmittance spectra of CuO thin films (Figure S5), where an LSPR band is visible at about 600 nm for Ar plasma treatment times longer than 90 s. Like Au and Ag, Cu also exhibits an LSPR band at visible frequencies [65,66]. Therefore, as the etching time increased, the Au NPs became more exposed to air, and the effective (“average”) RI of the surrounding dielectric medium (CuO matrix + air) decreased. This led to the narrowing, blue-shift, and transmittance increase in the LSPR band, as was already shown through theoretical investigations [28,34,55,67]. The same studies proved that such optical changes also lead to changes in the RIS of the films.

### 3.2. Gas Sensors Optimization—Gas Sensing Tests

**Figure 6:**
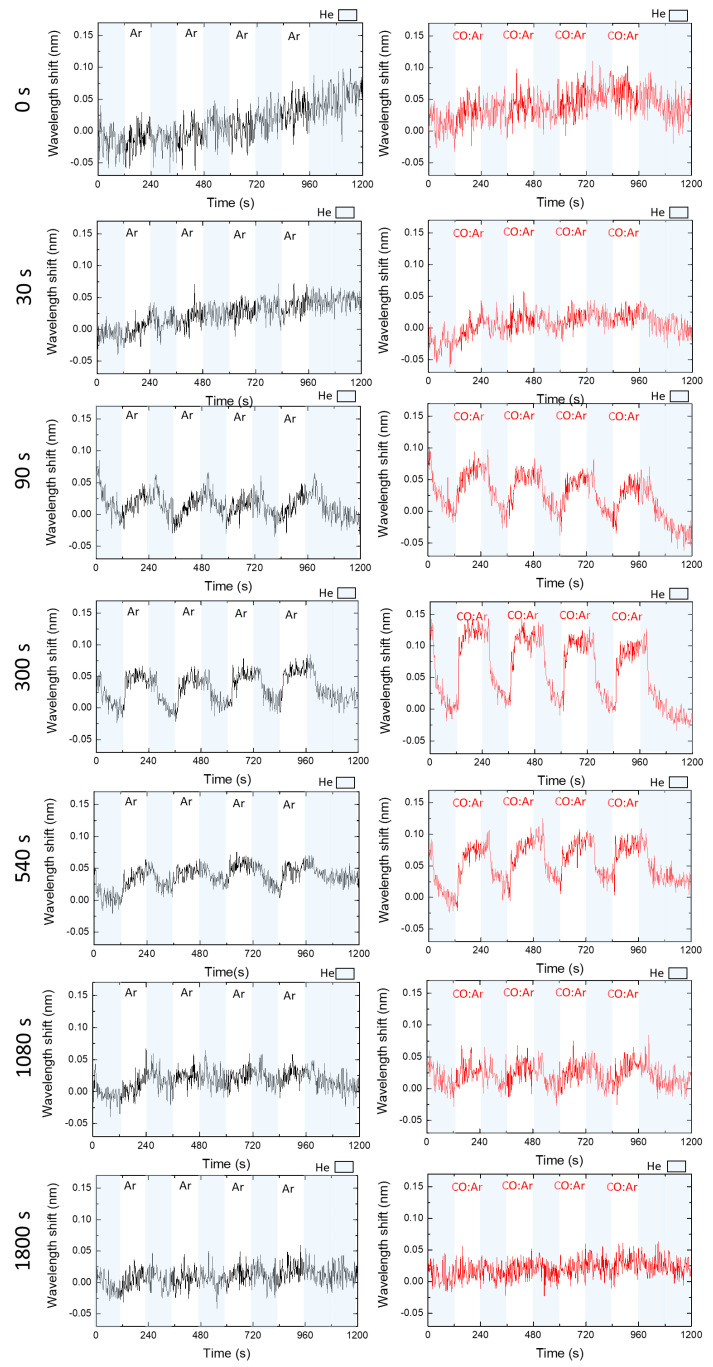
Wavelength shifts of the LSPR peak position of the Au:CuO film over time for 5 cycles of He (shaded areas) and pure Ar (black) and 50 ppm of CO in Ar (red) after different Ar plasma treatments times.

**Figure 7:**
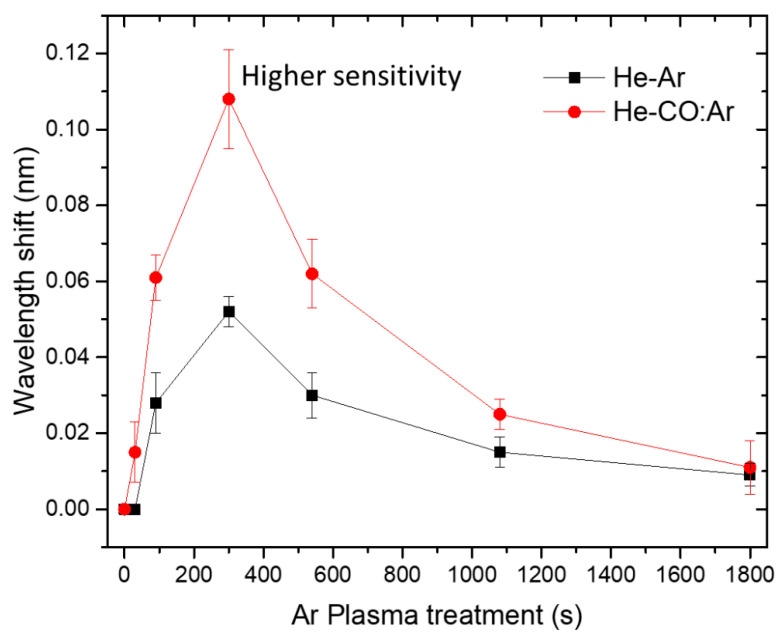
Average wavelength shift of the LSPR band of the Au:CuO film, as a function of the Ar plasma treatment, for 5 cycles of He-Ar and He-CO:Ar.

The gas sensitivity of the samples was evaluated for five cycles of He-Ar and He/CO:Ar at room temperature. The last cycle is the reference, where the measurement was made with two half-cycles of He. The wavelength peak position (minimum transmittance peak at the LSPR band) over time for the five cycles is plotted in Figure 6. The average wavelength shifts as a function of the Ar plasma treatment time for each He-Ar and He-CO:Ar test were processed by NANOPTICS software and are represented in Figure 7 and Table 2. As verified in previous works, the sensor did not show any response to both gases before the plasma treatment (t = 0 s) since the Au:CuO thin film surface was covered by a hydrocarbons layer, and the Au NPs near the film’s surface was surrounded by a sub-nanometric oxide layer as reported in previous work [62]. This limited the sensing volume of the NPs and the interaction of gas molecules with the CuO, which led to no gas detection.

After 30 s of plasma treatment, the peak wavelength red-shifted (Figure 6) about 0.015 ± 0.008 nm (Table 2) by changing the gaseous atmosphere to CO:Ar, returning to its baseline value after He reintroduction. The red-shift was even higher during CO:Ar exposure after 90 s of plasma treatment (0.061 ± 0.006 nm) and for Ar introduction (0.028 ± 0.008 nm) (Figure 6 and Table 2). Such results may correspond to changes in the RI of the bulk media from He to Ar and also CO:Ar since they have higher RIs than He. Here, according to the thin film characterization at 30 and 90 s of plasma treatment, the top layers were removed from the surface of the thin film, and the Au NPs were partially exposed, which allowed already monitoring of very slight LSPR band changes as the gas was switched.

The wavelength shift, i.e., the sensitivity, increased for longer plasma treatment times up to 300 s (Figure 7) to about 0.052 ± 0.004 nm in He/Ar cycles and 0.108 ± 0.013 nm in He/CO:Ar cycles (Table 2). However, for plasma treatments longer than 300 s, the wavelength shift and hence the sensitivity decreased with the increase in plasma treatment time (Figure 7). At 1800 s of plasma treatment, the wavelength shift was almost negligible (Table 2). Hence, at this time, it is possible to understand how the Au:CuO thin film properties correlate with gas detection. At the optimal plasma treatment time (t = 300 s), the CuO matrix was being etched and eroded, which led to a partial uncovering of the Au NPs and resulted in a maximum sensing response of the Au:CuO thin film. For plasma treatment times longer than 300 s, the presence of O-doped Cu nanostructures with sizes comparable to Au NPs was more and more evident due to the oxygen removal from the film’s surface, leading to the considerable reduction in the CuO matrix and consequent decrease in the gas sensing capabilities. From these results, it can be inferred that for a better performance of the sensor, the Au NPs should be partially exposed to the atmosphere, and the presence of the CuO matrix is also paramount to observing the LSPR band shifts. Apart from the stability and protection that CuO provides to the Au NPs, it also establishes an interaction between the gases and the thin films, increasing their RI sensitivity.

The wavelength shift for 50 ppm CO:Ar was always about two times higher than pure Ar (Table 2). The response of the first is much higher than expected for a simple RI change in the environment. This behavior might be associated with some adsorption of CO molecules by the CuO matrix, which results in an effective RI higher than that of the bulk gas. By taking this into account, the sensor RIS values for the different plasma treatment times were calculated using only the inert gas (Ar) tests by dividing the average wavelength shift of each test by the RI difference between Ar and He gases. At the optimal plasma treatment time (t = 300 s), the RIS of the system was about 849 ± 65 nm/RIU (Table 2), which corresponds to a two-fold improvement of the sensing response compared to the values obtained in the conditions of previous work [26]. This result demonstrates that the film has a good performance for sensing extremely small RI changes at room temperature when compared to the experimental and theoretical results from the literature, which reported sensitivities up to 1200 nm/RIU for Au NPs [28]. Furthermore, the SNR of the measurements also improved with increasing plasma treatment time until 300 s and worsened for longer plasma treatment times (Table 2). At 300 s of plasma treatment, the SNR for the He/Ar test was 3.2 and for the CO:Ar test was 6.7, which are values above the acceptable minimum value (SNR~3) for a measurement, also referred to as the confidence level [68].

## 4. Conclusions

The present study demonstrates how to achieve the maximum sensing response of a plasmonic Au:CuO thin film by applying different surface Ar plasma treatments, which modify the characteristics and properties of the thin film, and, consequently, its sensitivity to gas molecules.

It was concluded that the gas detection sensitivity improves with the plasma treatment time, being maximized at 5 min (300 s) of Ar plasma treatment. Here, a RIS of about 849 nm/RIU was estimated, which corresponds to a two-fold improvement in sensing response compared to previous work. In fact, up to 300 s of plasma treatment, the erosion of the CuO matrix, and consequent exposure of Au NPs to air lead to an LSPR band blue-shift and sensing response increase. However, the gas detection capabilities of the sensor decrease for longer plasma treatment times. The metal-oxide bonds are broken, and oxygen is removed from the film’s surface, leading to the formation of O-doped Cu nanostructures and a consequent decrease in gas sensitivity. It can then be inferred that the CuO matrix presence in the LSPR sensor plays a decisive role not only in providing stability and protection to the Au NPs but also in allowing interactions between the thin film’s surface and the gases, improving their RI sensitivity.

In conclusion, the results obtained demonstrate that for better sensor performance, the Au:CuO thin film should be subject to low-power Ar plasma treatment for 5 min. Under these conditions, an optimal design was attained, where the Au NPs are partially exposed to the free space while the host CuO matrix is still partially embedding them and contributing to an optimized sensitivity. The potential of this system to be employed as an optical (LSPR) CO sensor at room temperature was demonstrated, although further research is needed to better attest to the selectivity of the sensor. Therefore, gas sensing tests in the presence of other pollutant atmospheres should be performed, and the immobilization of recognition elements to increase the selectivity will be a pursuit in the near future.

From the Ar plasma treatments and LSPR gas sensing, it can be concluded that the optimization of the Au:CuO thin film system was successful. The maximum sensing response was achieved after 5 min of Ar plasma treatment, and the Au:CuO thin film can be further exploited as a gas sensor or even as an LSPR platform for label-free biosensors.

## Figures and Tables

**Table 1:** Surface properties of the Au:CuO thin films extracted from AFM and SEM studies after different plasma treatment times.

Time of Ar Plasma Treatment (s)	AFM 5 × 5 µm			SEM			
	Ra (Sa) (nm)	RMS (Sq) (nm)	<Height> (nm)	NPs Count	<Feret Diameter> (nm)	<Nearest Neighbor> (nm)	Av. Aspect Ratio
0	5.4	7.0	56.1	120	51 (σ = 29)	47 (σ = 19)	1.35 (σ = 0.5)
30	4.4	6.0	59.9	128	48 (σ = 28)	46 (σ = 19)	1.43 (σ = 0.6)
90	5.0	6.6	61.9	179	41 (σ = 26)	38 (σ = 14)	1.46 (σ = 0.9)
300	6.9	8.6	69.2	145	48 (σ = 25)	43 (σ = 15)	1.36 (σ = 0.6)
540	12.5	15.4	115.0	133	50 (σ = 30)	40 (σ = 19)	1.46 (σ = 0.5)
1080	13.1	16.1	119.8	142	46 (σ = 27)	44 (σ = 15)	1.58 (σ = 1)
1800	12.2	15.1	117.2	143	45 (σ = 27)	42 (σ = 18)	1.46 (σ = 0.6)

**Table 2:** Optical response parameters of the He-Ar and He-CO:Ar tests as a function of Ar plasma treatment time.

Time of Ar Plasma Treatment (s)	Average Wavelength Band Shift (nm) He-Ar	SNR He-Ar	Average Wavelength Band Shift (nm) He-CO:Ar	SNR He-CO:Ar	RIS (He-Ar)
0	0	0	0	0	0
30	0	0	0.015 ± 0.008	1.4	0
90	0.028 ± 0.008	1.8	0.061 ± 0.006	3.9	457 ± 130
300	0.052 ± 0.004	3.2	0.108 ± 0.013	6.7	849 ± 65
540	0.03 ± 0.006	2.3	0.062 ± 0.009	3.6	490 ± 98
1080	0.015 ± 0.004	1.3	0.025 ± 0.004	1.6	245 ± 65
1800	0.009 ± 0.003	1.1	0.011 ± 0.007	1.1	147 ± 49

## Data Availability

Not applicable.

## Supplementary Materials

The following supporting information can be downloaded at: https://www.mdpi.com/article/10.3390/s22187043/s1, Figure S1: Photographs of (a) Low-Pressure Plasma Cleaning equipment used for film’s surface modification and (b) custom-made high-resolution LSPR spectroscopy system for gas sensing test measurements in a controlled atmosphere. Figure S2: RBS spectrum curve fitting with simulations of the Au:CuO thin film for different plasma treatment times. The original spectrum is represented by red round dots and the simulation curve by solid black line. The spectra show that after 540 s of plasma treatment, there is a dramatic decrease in O content, followed by a considerable increase in the surface roughness and measurement uncertainty; Figure S3: X-ray diffraction patterns of the CuO thin films after different Ar plasma treatment times. The diffraction patterns confirmed the amorphous phase of the CuO thin films. After 540 s of Ar plasma treatment, a peak with low intensity at 2θ = 43.6° is shown, which might correspond to the (111) plane of the FCC structure of metallic Cu.; Figure S4: Raman spectra of the CuO thin films after different Ar plasma treatment times. The peak observed before the plasma treatments (t = 0 s) is assigned to the principal Raman active mode of CuO, located at 290 cm^−1^. This result indicates the presence of a CuO phase until 90 s of plasma treatment. After 300 s of plasma treatment, no peak is detected; Figure S5: Two-dimensional AFM height images (a), height distribution profiles (AFM analysis using the Gwyddion software) (b) and surface mean roughness (Sa), root mean square roughness (Sq) and mean height (<height>) (c) of the CuO thin films as a function of Ar plasma treatment time; Figure S6: Transmittance spectra of the CuO thin films after different etching times with Ar plasma treatments. The presence of an LSPR band at about 600 nm for Ar plasma treatment times longer than 90 s confirms the formation of O-doped Cu nanostructures after 300 s of plasma treatment.
