# Supplementary material for: Optimization of Au:CuO Thin Films by Plasma Surface Modification for High-Resolution LSPR Gas Sensing at Room Temperature
Source: Sensors (Basel). 2022 Sep 17;22(18):7043. doi: 10.3390/s22187043 (PMC9501632; doi:10.3390/s22187043)
Supplement: Supplementary file 1 [file sensors-22-07043-s001.zip › sensors-1919846-supplementary.pdf]

# Optimization of Au:CuO thin films by plasma surface modification for high-resolution LSPR gas sensing at room temperature


Manuela Proença[1], Marco S. Rodrigues[1], M. Cidalia R. Castro[2], Pedro V. Rodrigues[2], Ana V. Machado[2], Eduardo Alves[3], Nuno P. Barradas[4], Joel Borges[1*], Filipe Vaz[1]

*[1]Physics Center of Minho and Porto Universities (CF-UM-UP), University of Minho, Campus de Azurém, 4800-058 Guimarães, Portugal*

*[2]Instituto de Polímeros e Compó sitos, Universidade do Minho, Campus de Azurém, 4800-058 Guimarães, Portugal*

*[3]IPFN, Instituto de Plasmas e Fusão Nuclear, Instituto Superior Técnico, Universidade de Lisboa, Estrada Nacional 10 Bobadela LRS, 2695-066, Portugal*

*[4]Centro de Ciências e Tecnologias Nucleares, Instituto Superior Técnico, Universidade de Lisboa, Estrada Nacional 10 Bobadela LRS, 2695-066, Portugal*

[*]Corresponding author: Joel Borges ; joelborges@fisica.uminho.pt; joelborges22@gmail.com ; +351 253 510 471

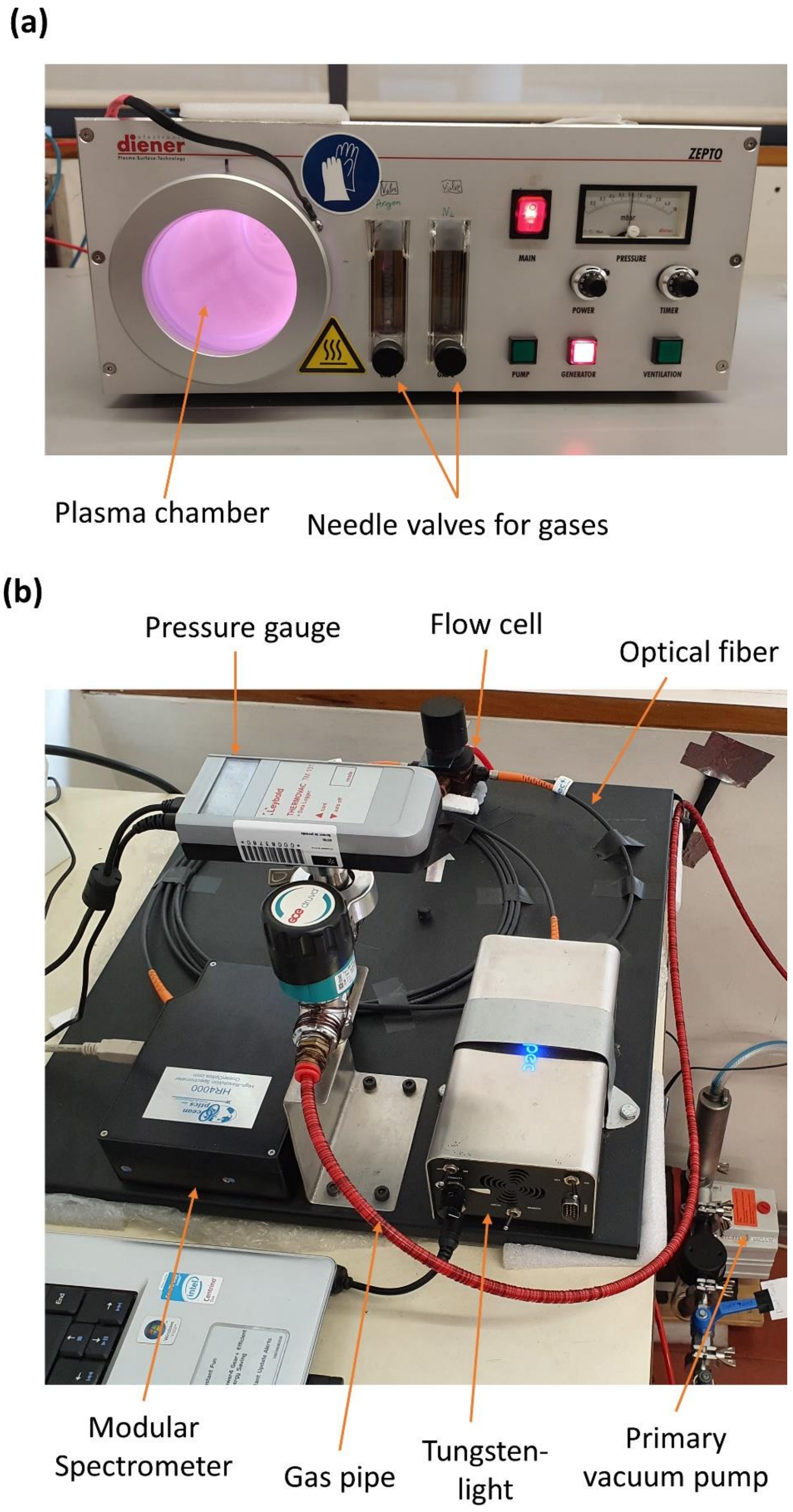


**Figure S1:** Photographs of (a) Low-Pressure Plasma Cleaning equipment used for film's surface modification, and (b) custom-made high-resolution LSPR spectroscopy system for gas sensing test measurements in a controlled atmosphere.

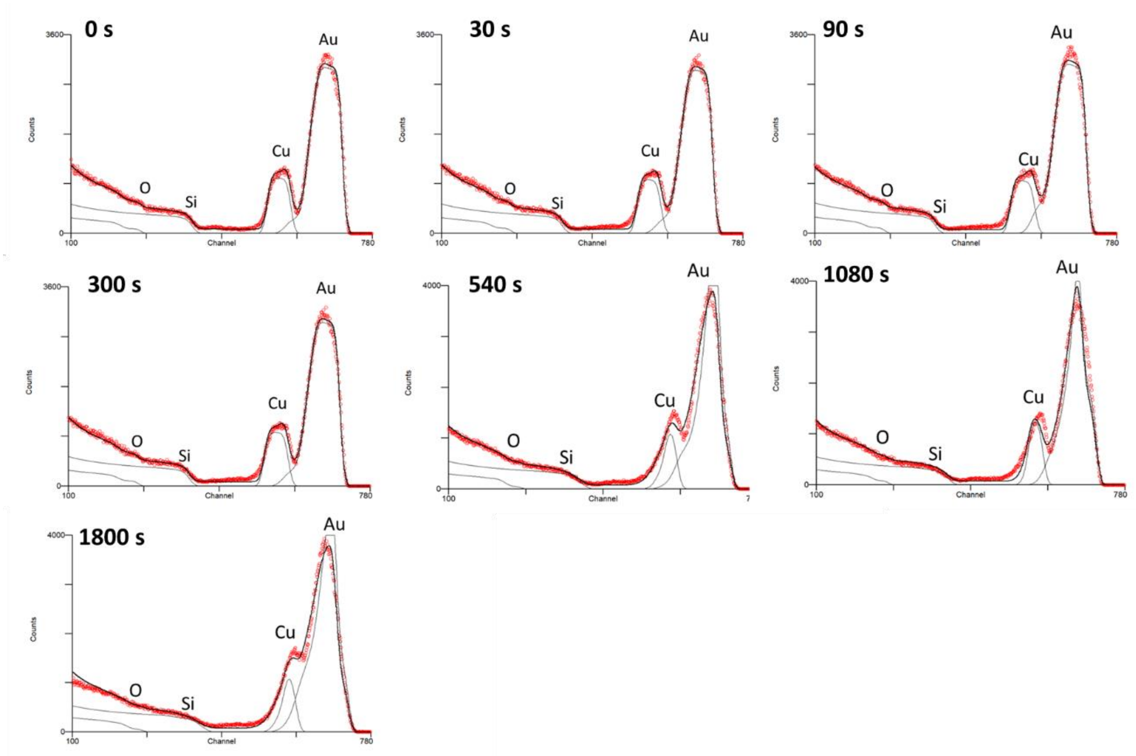


**Figure S2:** RBS spectrum curve fitting with simulations of the Au:CuO thin film for different plasma treatment times. The original spectrum is represented by red round dots and the simulation curve by black solid line. The spectra show that after 540 s of plasma treatment, there is a dramatic decrease in O content, followed by a considerable increase in the surface roughness and measurement uncertainty.

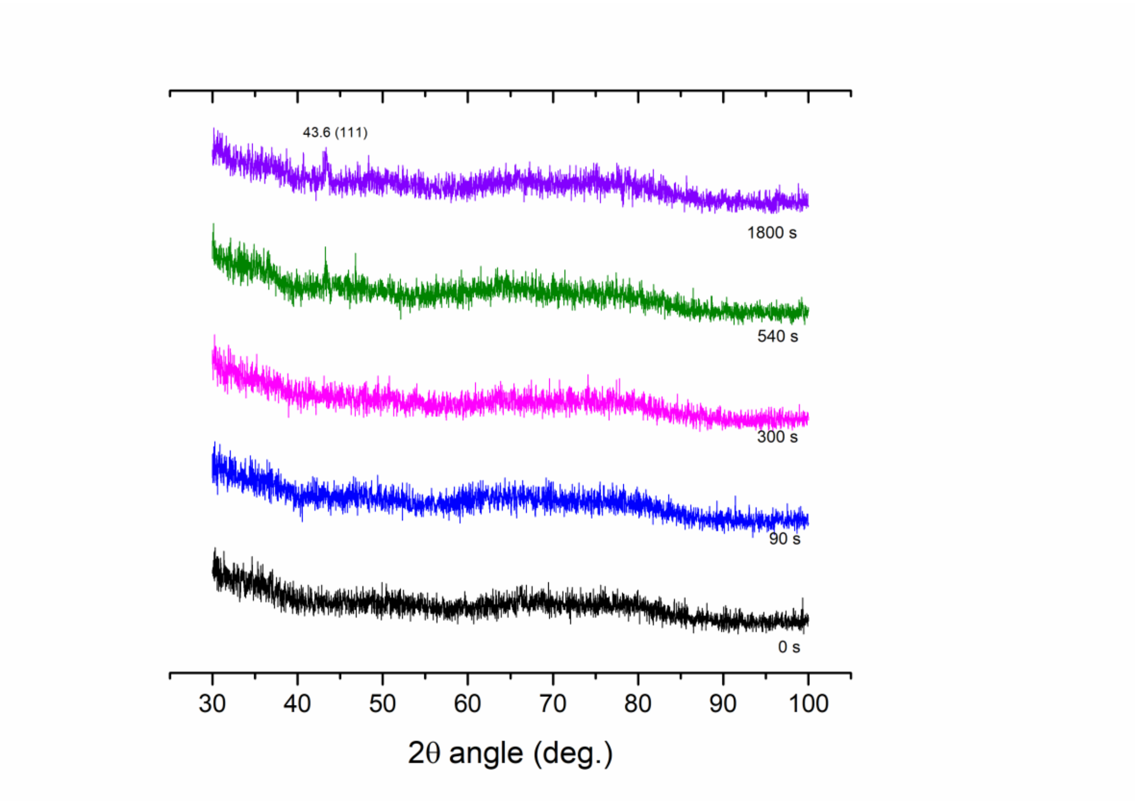


**Figure S3:** X-ray diffraction patterns of the CuO thin films after different Ar plasma treatment times. The diffraction patterns confirmed the amorphous phase of the CuO thin films. After 540 s of Ar plasma treatment, a peak with low intensity at $2\theta = 43.6°$ is shown, which might correspond to the (111) plane of the FCC structure of metallic Cu.

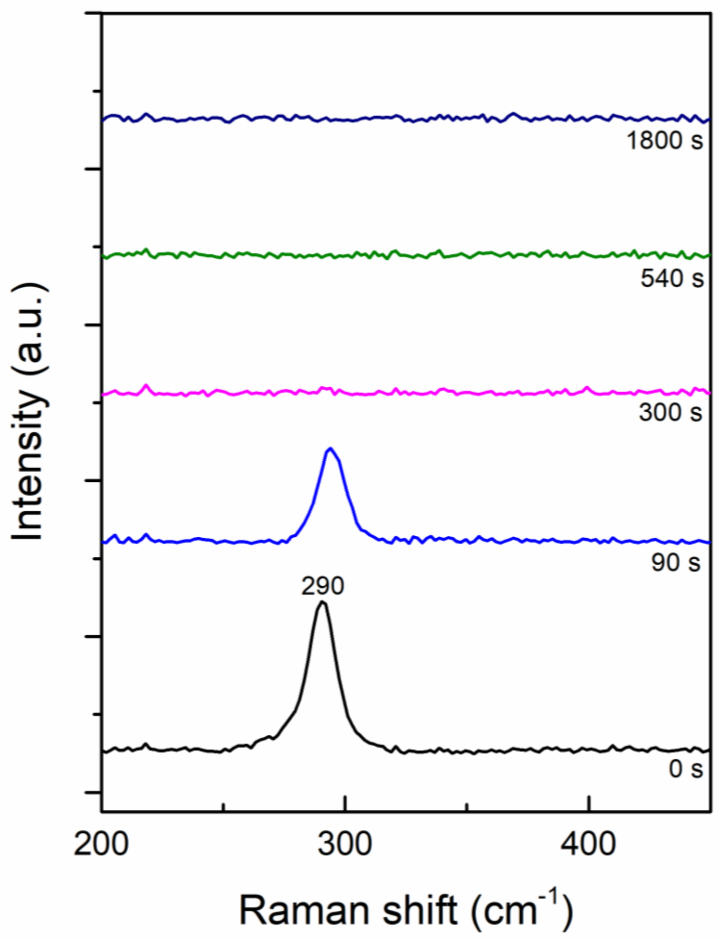


**Figure S4:** Raman spectra of the CuO thin films after different Ar plasma treatment times. The peak observed before the plasma treatments (t = 0 s) is assigned to the principal Raman active mode of CuO, located at 290 $cm^{-1}$. This result indicates the presence of a CuO phase until 90 s of plasma treatment. After 300 s of plasma treatment, no peak is detected.

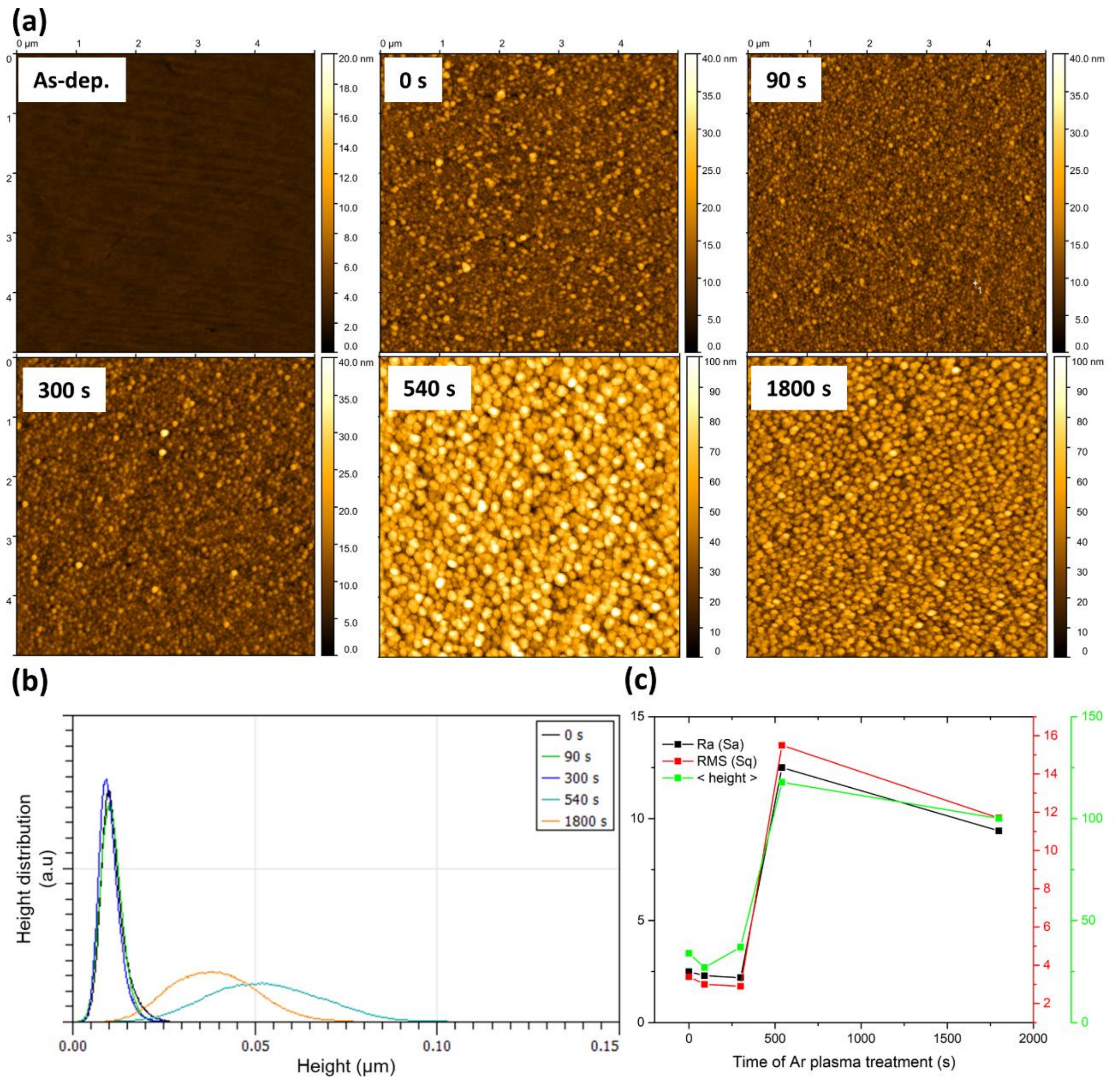


**Figure S5:** 2D AFM height images **(a)**, height distribution profiles (AFM analysis using the Gwyddion software) **(b)** and surface mean roughness (Sa), root mean square roughness (Sq) and mean height (< height > ) **(c)** of the CuO thin films as a function of Ar plasma treatment time.

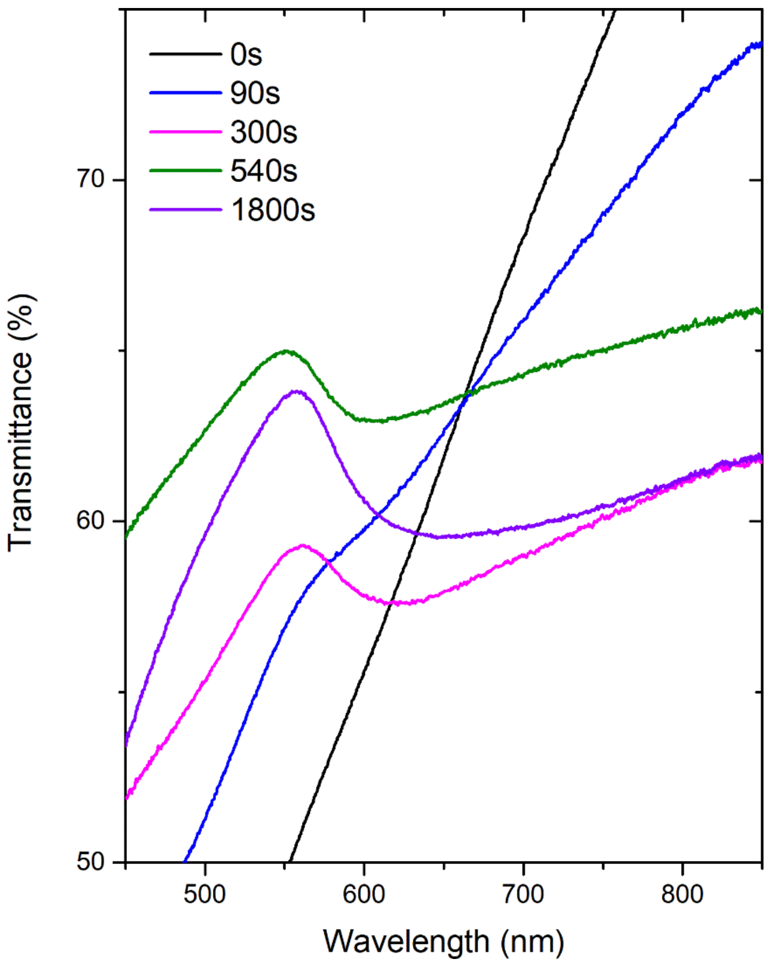


**Figure S6:** Transmittance spectra of the CuO thin films after different etching times with Ar plasma treatments. The presence of an LSPR band at about 600 nm for Ar plasma treatment times longer than 90 s confirms the formation of O-doped Cu nanostructures after 300 s of plasma treatment.
